# Supplementary material for: Production of Ultrathin and High-Quality Nanosheet Networks via Layer-by-Layer Assembly at Liquid–Liquid Interfaces
Source: ACS Nano. 2024 Nov 13;18(47):32589–601. doi: 10.1021/acsnano.4c09745 (PMC11603785; doi:10.1021/acsnano.4c09745)
Supplement: Supplementary file 1 — nn4c09745_si_001.pdf [file nn4c09745_si_001.pdf]

Production of Ultra-Thin and High-Quality Nanosheet Networks via Layer-by-Layer Assembly at Liquid-Liquid Interfaces

Joseph Neilson[1], Eoin Caffrey[1], Oran Cassidy[1], Cian Gabbett,[1] Kevin Synnatschke[2], Eileen Schneider[3], Jose Maria Munuera[4], Tian Carey,[1] Max Rimmer[5], Zdeněk Sofer[6], Janina Maultzsch[3], Sarah J. Haigh[5] and Jonathan N. Coleman[1]*

[1]*School of Physics, CRANN & AMBER Research Centres, Trinity College Dublin, Dublin 2, Ireland*

[2]*Faculty of Chemistry and Food Chemistry, Dresden University of Technology, 01062 Dresden, Germany*

[3]*Department of Physics, Friedrich-Alexander-Universität, Erlangen-Nürnberg, Staudtstr. 7, 91058 Erlangen, Germany*

[4] *Department of Physics, Faculty of Sciences, University of Oviedo, C/ Leopoldo Calvo Sotelo, 18, 33007 Oviedo, Asturias, Spain.*

[5] *Department of Materials and National Graphene Institute, The University of Manchester, Oxford Rd, Manchester, M13 9PL, UK*

[6] *Department of Inorganic Chemistry, University of Chemistry and Technology Prague, Technická 5, 166 28 Prague 6, Czech Republic*

*colemaj@tcd.ie (Jonathan N. Coleman); Tel: +353 (0) 1 8963859.

In this document:

T1: Literature examples of graphene conductive networks

T2: Literature examples of MoS2 TFTs

T3: Literature examples of silver conductive networks

Supplementary figures

Examples of measured dielectrically gated transfer curves

Removal of Rc effects from MoS_2 TFTs

Table 1: Literature examples of graphene conductive networks

**Ref.**	**Graphene type**	**Deposition method**	**Anneal Temp (°C)**	**other treatments**	**Conductivity (S/m)**	**Thickness (m)**	**Optical transmittance, T**
1	EE	Inkjet	300		2.5×10^3	4.45×10^{-7}	0.53
2	EE	vacuum filtration	n/a	Nitric acid	1.3×10^3	n/a	N/A
3	EE	vacuum filtration	600		8.6×10^4	3.6×10^{-8}	0.05
4	EE	Sprayed	500	vacuum	4.7×10^4	6.5×10^{-7}	
5	EE	Inkjet	300		3.91×10^4	2.23×10^{-7}	
6	shear exfoliation	Inkjet	350		4.0×10^4	1.0×10^{-7}	0.86
7	LPE	Inkjet	450		6.25×10^4	4.8×10^{-7}	
8	LPE	screen printing	100	calendaring	7.13×10^4	*7.8×10^{-6}	
9	LPE	sprayed	270		1.2×10^5	5×10^{-7}	

Table 2: MoS_2 TFTs literature examples.

Ref	MoS2 type	Gate	Deposition method	Max Anneal Temp (°C)	Other treatments	Mobility (cm^2/Vs)	on/off	Thickness (nm)
[10]	EE	SiO2	Liquid interface deposition	200		0.73	10^5	2
[11]	EE	$Sr_{1.8}Bi_{0.2}Nb_3O_{10}$	Spin coating	250	TFSI in DCE at 80 C, e-beam irradiation at 1600 kGy	10	10^6	7
[12]	LPE	EMI-TFSI	Drop cast	90	BDT crosslinking	10	10^4	700
[13]	EE	SiO2	Spin coating	200	TFSI in DCE at 80 C	11	10^6	10
[14]	EE	AlOx	Inkjet	400	TFSI in DCE at 100 C for 1h	0.1	50	20
[15]	EE	EMIM-TFSI	Langmuir-Schaefer	120		11	2000	25

Table 3: Literature examples of Ag nanoparticle conductive networks

**Ref**	**Ag type**	**Deposition Method**	**Anneal Temp (°C)**	**Conductivity (S/m)**	**Network Thickness**
[16]	AgNS	Direct Write	200	1.3×10^{7}	
[17]	AgNP	Drop cast	300	5.0×10^{6}	N/A
[17]	AgNW	Drop cast	70	5.56×10^{6}	N/A
[17]	AgNS	Drop cast	300	2.4×10^{5}	N/A
[18]	AgNF	Modified gel pen	200	1.1×10^{7}	~200 µm
[19]	AgNS	Thermal inkjet	200	6.0×10^{6}	200 nm
[20]	AgNP	Inkjet	250	2.5×10^{7}	0.2 - 08 µm

Supplementary Figures

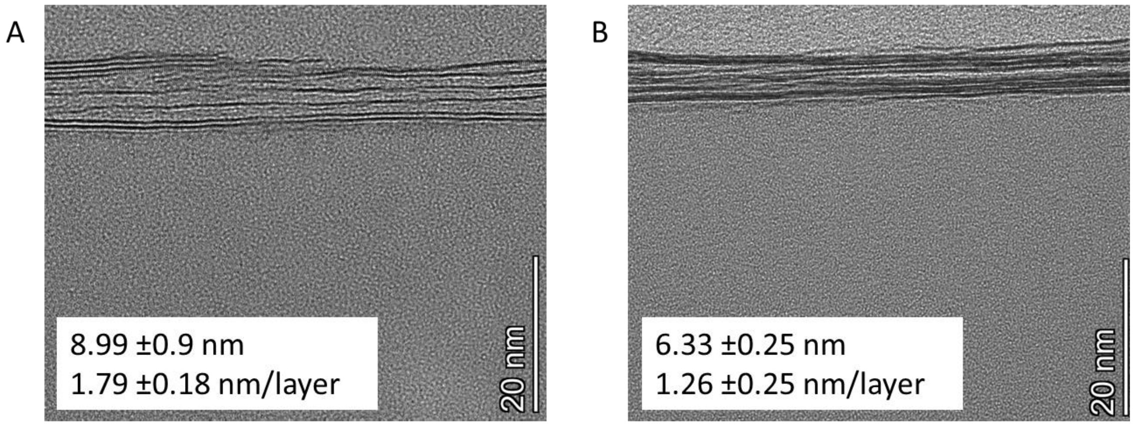

Figure S1: TEM x-section image of MoS_2 5L film A. Without the IPA washing step between each deposition B. with the IPA washing step between each deposition. The reduced interlayer distance is as a result of removing polymer from the surface of each layer.

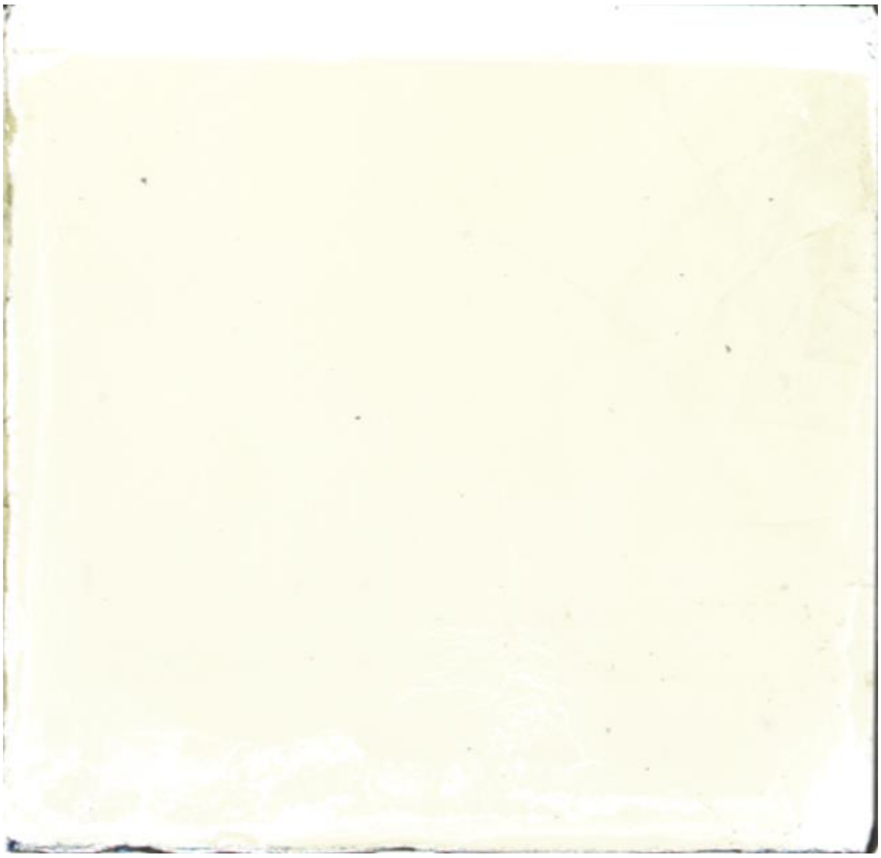

Figure S2: Typical image of a liquid interface deposited film taken by the scanner. Shown here is a 1L MoS_2 film. The blank substrate area at the top of the image was used to calibrate the pixel intensity to a thickness of zero.

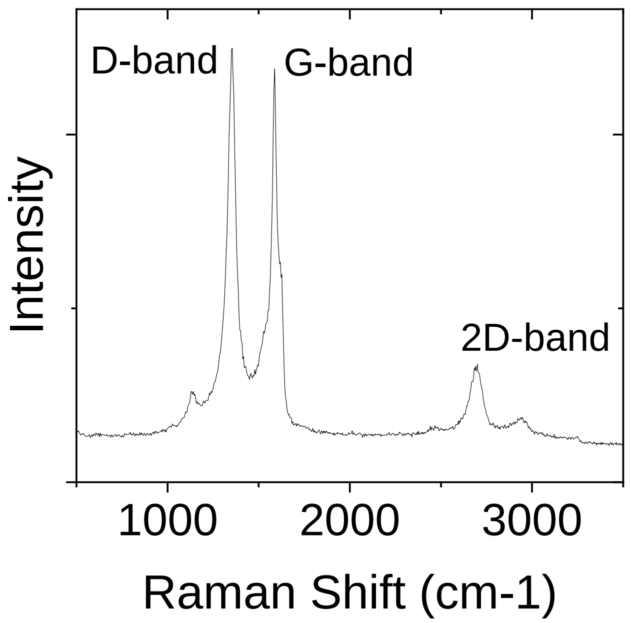

Figure S3. Typical Raman spectra for a 1L EE graphene network deposited on quartz. The intense D-band highlights the defective nature of our EE graphene nanosheets.

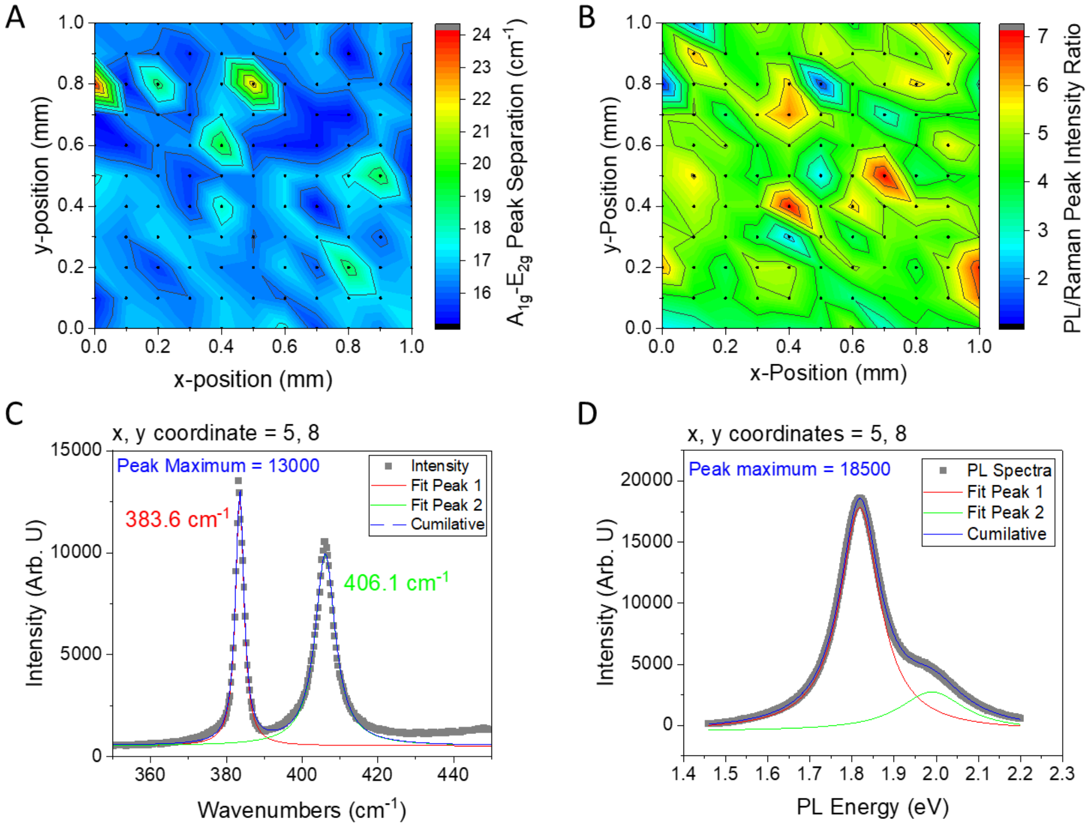

Figure S4: MoS_2 monolayer film Raman and PL spectroscopic mapping. A. $A_{1g} - E_{2g}$ Raman peaks separation map and B. PL/Raman peak intensity ratio maps obtained from a MoS_2 1L film. In A and B, each measurement spot is ~ 1um in diameter, and the distance between two neighbouring spots is ~100um. The 2D contour plots have been interpolated between datapoints. C. Example Raman spectra (grey squares) from the x, y coordinate = 5, 8 (x, y position = 0.5, 0.8 mm). Included in the plot are Lorentzian fits of the E_{2g} (red trace) and A_{1g} (green trace) peaks. The blue trace is the cumulative fit to the spectra. The peak positions of the Lorentzian fits are used to calculate the peak separation values in A. D is an example PL spectra from the same position as C (x, y coordinate = 5, 8). The peak maximums from the cumulative fits (blue trace) in C and D are used to calculate the PL/Raman peak intensity values in B (PL/Raman Intensity = PL cumulative peak maximum/Raman cumulative peak maximum.

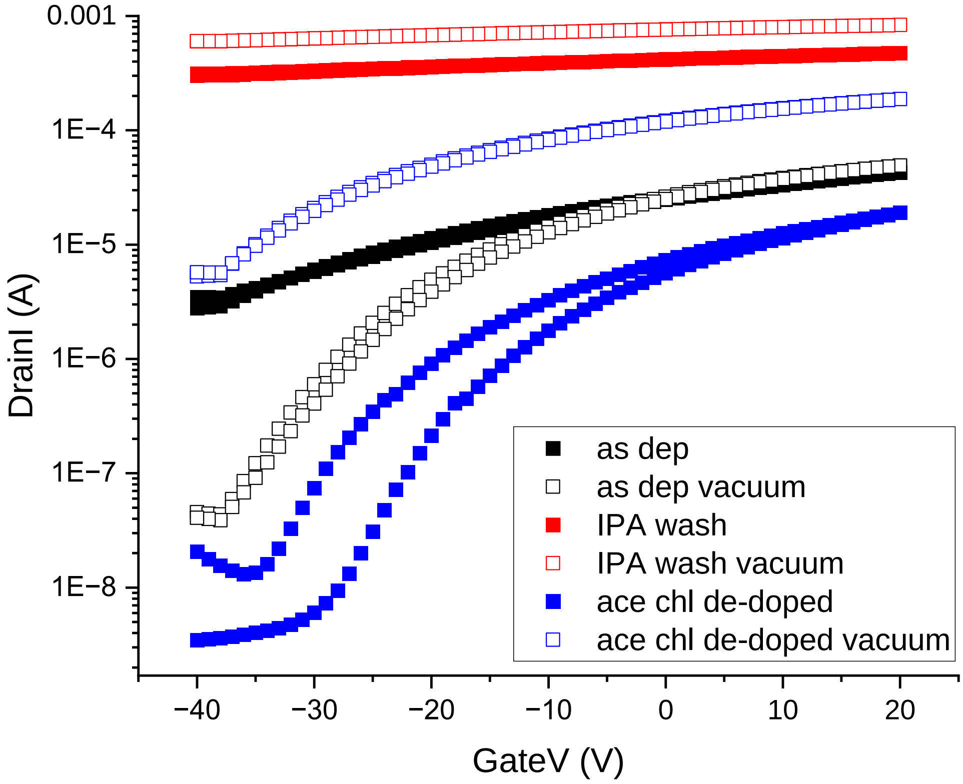

Figure S5: Effect of IPA washing on MoS_2 TFTs. The as-deposited MoS_2 TFT with moderate on-off current ratio (black datapoints) was first washed with IPA at 80 °C (red datapoints), doping the MoS_2,[21] and resulting in an increased carrier density and reduced on-off current ratio. The doped MoS_2 device can then be de-doped by sequential soaking in acetone for 20 minutes followed by chloroform for 20 minutes (blue datapoints), reducing the carrier density and increasing the on-off current ratio.

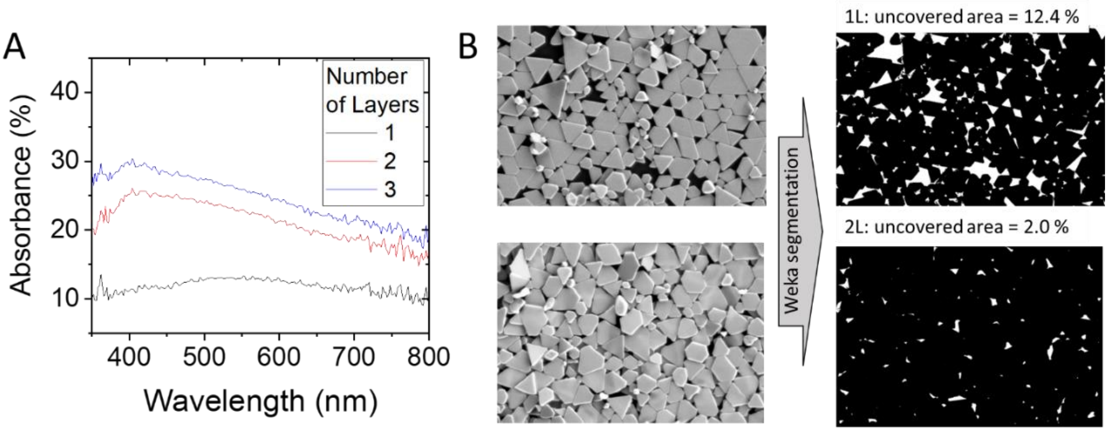

Figure S6: A. Absorbance spectra for AgNS 1L, 2L and 3L networks, showing a ~10 % increase in absorbance between 1L and 2L networks. B. Statistical analysis of network fill factor in 1L And 2L AgNS networks, obtained by processing the image using the Weka Segmentation package of the FIJI image processing software.[22]

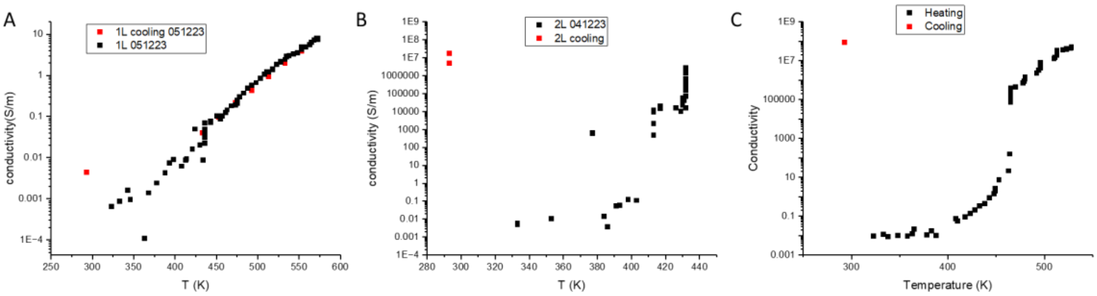

Figure S7: Conductivity/temperature plots for A. 1L, B. 2L and C. 3L AgNS networks upon heating to sintering temperature and cooling. The conductivity of the 1L film does not improve due to lack of sintering, as discussed in the main manuscript.

Examples of measured transfer curves.

For each network thickness (1L, 2L, 3L, 4L, 5L), dielectrically gated transfer curves were measured for 4 nominally identical channels for each channel length. As shown in the main text, these curves only cover a portion of the switching range due to the thickness of the dielectric used here (230 nm). Shown in figure S8A are example curves for each network thickness.

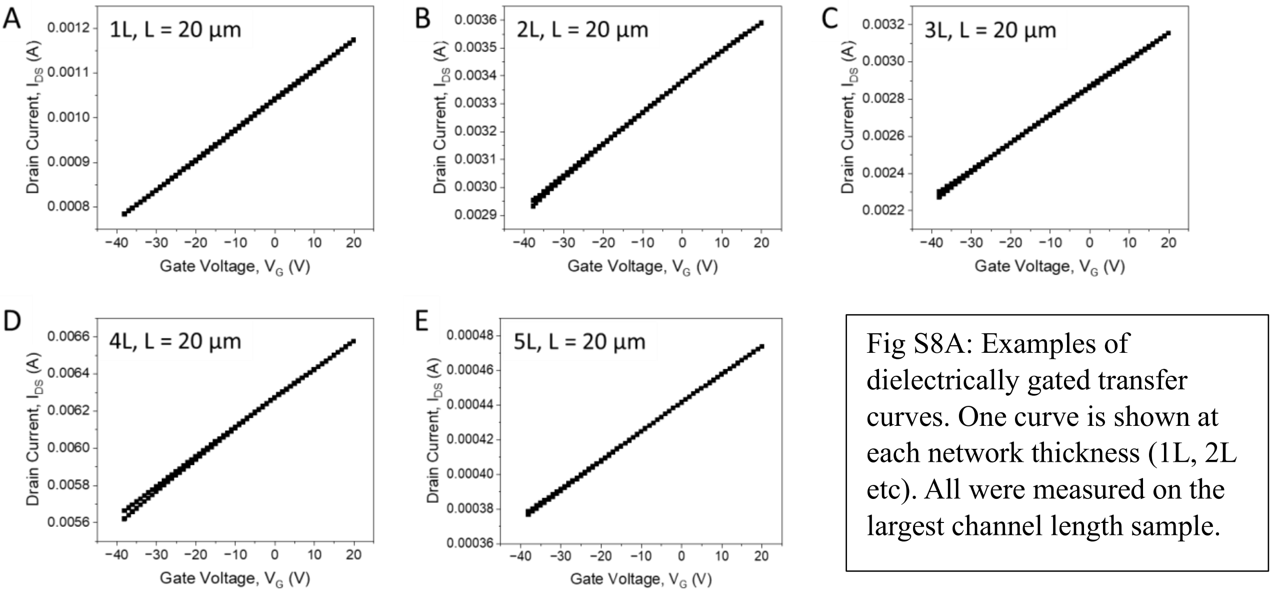

Fig S8A: Examples of dielectrically gated transfer curves. One curve is shown at each network thickness (1L, 2L etc). All were measured on the largest channel length sample.

For three network thicknesses (1L, 2L, 3L), electrochemically gated transfer curves were measured. Shown in figure S8B are example curves for each network thickness.

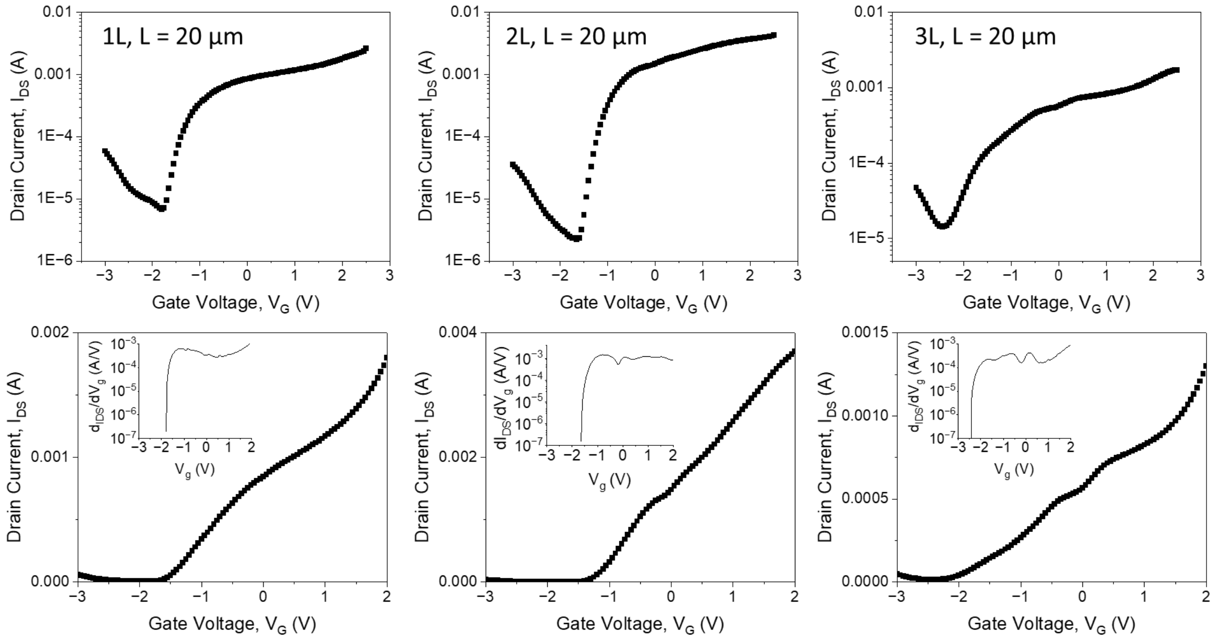

Fig S8B: Examples of electrochemically gated transfer curves. One curve is shown at each network thickness (1L, 2L, 3L). All were measured on the largest channel length sample. The top row shows the transfer curves with the current on a log scale while the bottom row shows a linear scale. The insets of the bottom row show the transconductance plotted versus gate voltage.

Correcting conductivity and mobility for the effects of contact resistance

Here we apply four methods to find corrected conductivity and mobility values where the effects of contact resistance have been removed.

1: Comparing sheet resistance values obtained from van der Pauw (vdP) and network resistance v channel length measurements (TLM)

In order to confirm the close agreement between linear four-point probe and van der Pauw measurements, we made a comparison for one sample. For a 6 layer graphene network, the sheet resistance was measured using 4-point probe techniques in linear, 4-pp (spacing: 1.27 mm) and van der Pauw, VDP contact arrangement. The table above shows that the methods result in sheet resistance values which are equivalent, within error of the measurement. For the van der Pauw arrangement, standard deviation was taken from two measurements in perpendicular orientation, such that $R_1 = \frac{I_{2,4}}{V_{1,3}}$ and $R_2 = \frac{I_{3,4}}{V_{1,2}}$ are the measured resistances in each orientation. This was converted to sheet resistance as $R_S = R \times \frac{\pi}{\ln 2}$. The average sheet resistance and standard deviation in linear 4-point probe method was obtained by measuring 4-pp resistance in three random positions across a 2.5cm×2.5cm network.

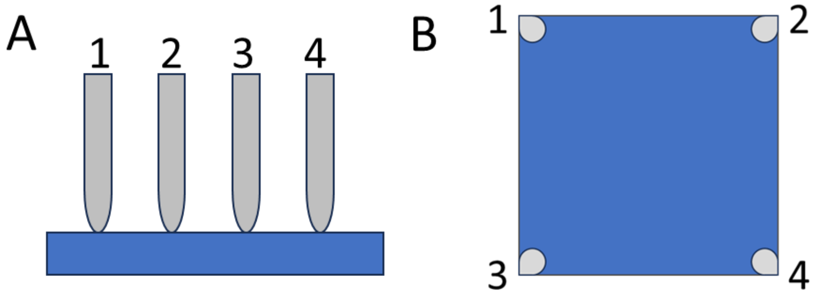

C

Layer number	Measurement method	Rs Average (Ohm/sq)	Rs_SD
6	VDP	1558	59.7
6	4-pp	1599	12.8

Figure S9: Sheet resistance measurements. A. Linear 4-point probe method, 4-pp whereby current is sourced between the outer electrodes 1, 4 and voltage is measured between the inner electrodes 2, 3. B. Van der Pauw arrangement, VDP where current is sourced across adjacent electrodes 1, 3 and voltage is measured across opposite electrodes 2, 4. C. The average and standard deviation of sheet resistance for a 6 layer graphene network, measured using VDP and 4-pp methods.

2: Extracting contact resistance (R_C) from network resistance v channel length measurements (TLM)

The measured resistance (R_A) of a device, consisting of a channel material contacted with electrodes is not always equal to the actual resistance of the channel material alone. This is because R_A contains contributions from the material resistance and the contact resistance. Thus, R_A can be considered an apparent resistance. If it is directly converted to a conductivity using the material length (L), width (w) and thickness (t), this will yield an "apparent conductivity", $\sigma_A = \frac{1}{R_A}\frac{L}{wt}$, i.e. one which is not corrected for the effects of contract resistance.

The effects of contact resistance must be considered if one is to convert the measured (apparent) resistance, R_A, of a sample to the real material conductivity, σ_M. For the purposes of clarity, here in the SI, we will refer to the real material conductivity as σ_M and below to the real material mobility as μ_M. However, in the main text, these parameters will be referred to as σ_{Net} and μ_{Net} to distinguish them from the nanosheet conductivity and mobility.

The measured (apparent) resistance, R_A, of a network versus the channel length, L, is given by

$$R_A = 2R_C + \frac{1}{\sigma_M}\frac{L}{wt}$$

where R_c is contact resistance at each electrode, w is channel width, t is film thickness. This means that measurements of R_A at different channel lengths (L) can be used to obtain both R_C and σ_M. These are called transfer length method (TLM) measurements.

All of our MoS2 networks were deposited on commercial FET test chips from Fraunhofer IPMS which allowed measurements of resistance for different channel lengths (see Figure S10).

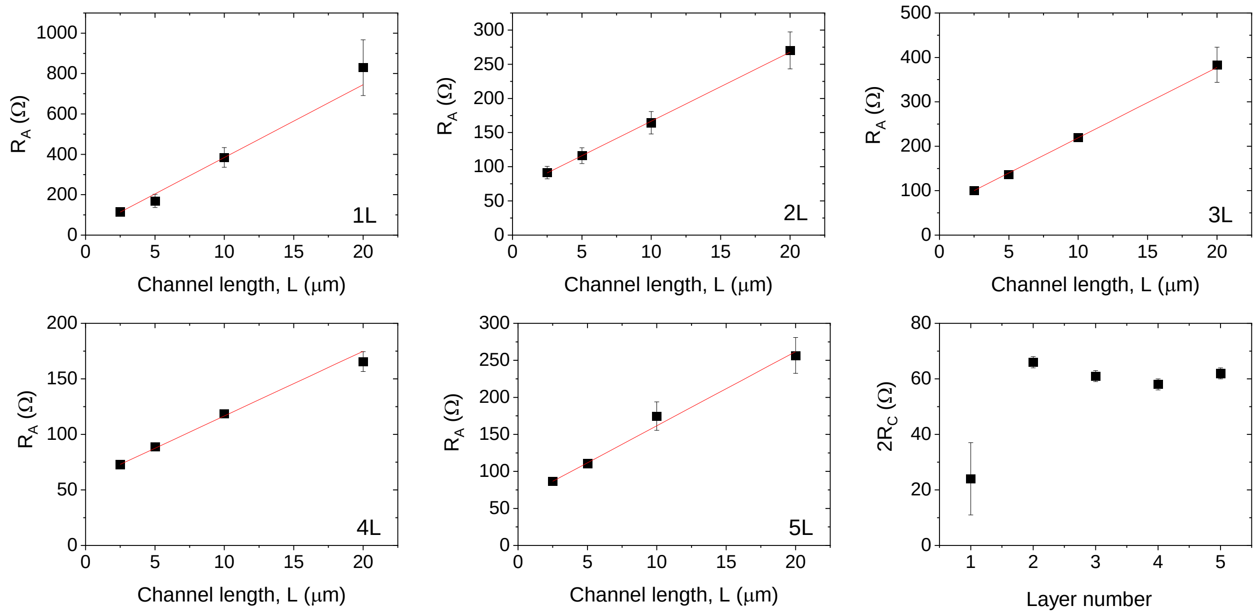

Figure S10: Resistance of MoS2 networks, plotted as a function of channel length for various network thicknesses (1L, 2L, 3L, 4L, 5L). These measurements were made on commercial FET test chips from Fraunhofer IPMS (Figure S11). Bottom right, the resultant contact resistance plotted versus film thickness expressed as layer number.

We note that the TLM method requires that only the channel length, L is varied, while other parameters, such as channel width, W and contact material are held constant. Hence, throughout this work, TLM was only performed on commercial FET test chips from Fraunhofer IPMS. These chips have prepatterned electrode arrays, which have fixed channel width of either 2 mm (Gen 5) or 10 mm (Gen 4). An optical microscope image of an FET test chip is provided in figure S11.

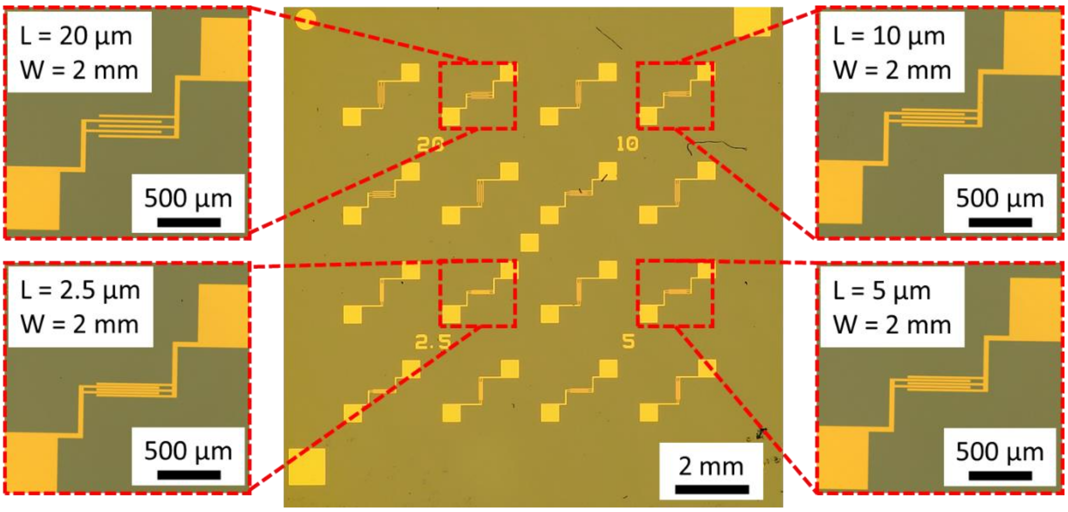

Figure S11: Optical image of commercial FET test chips from Fraunhofer IPMS.

3: Correcting network mobility for the effects of contact resistance (method 1)

The mobilities measured in the TFT experiments are also affected by the contact resistance and so must be considered as "apparent mobilities". Thus, they need to be corrected.

The equation given above (i.e. $R_A = 2R_C + \frac{1}{\sigma_M}\frac{L}{wt}$) can be combined with $R_A = \frac{1}{\sigma_A}\frac{L}{wt}$ to give a relationship between apparent conductivity and real material conductivity:

$$\sigma_A = \frac{\sigma_M}{1+\frac{2R_C wt\sigma_M}{L}}$$

We can then convert the apparent and material conductivities to the apparent (μ_A) and material (μ_M) mobility using $\sigma_A = ne\mu_A$ and $\sigma_M = ne\mu_M$. This yields an equation relating the measured, apparent mobility (μ_A), which includes the effects of contact resistance to the actual material mobility (μ_M) which does not include the effects of contact resistance:

$$\mu_A = \frac{\mu_M}{1+2R_c newt\mu_M / L}$$

This means that measurements of μ_A at different channel lengths (L) can be fit using this equation to obtain μ_M.

Our data for measured, apparent mobility is plotted below as a function of channel length for IPA-washed MoS2 networks of different thickness (layer number e.g. 1L, 2L etc).

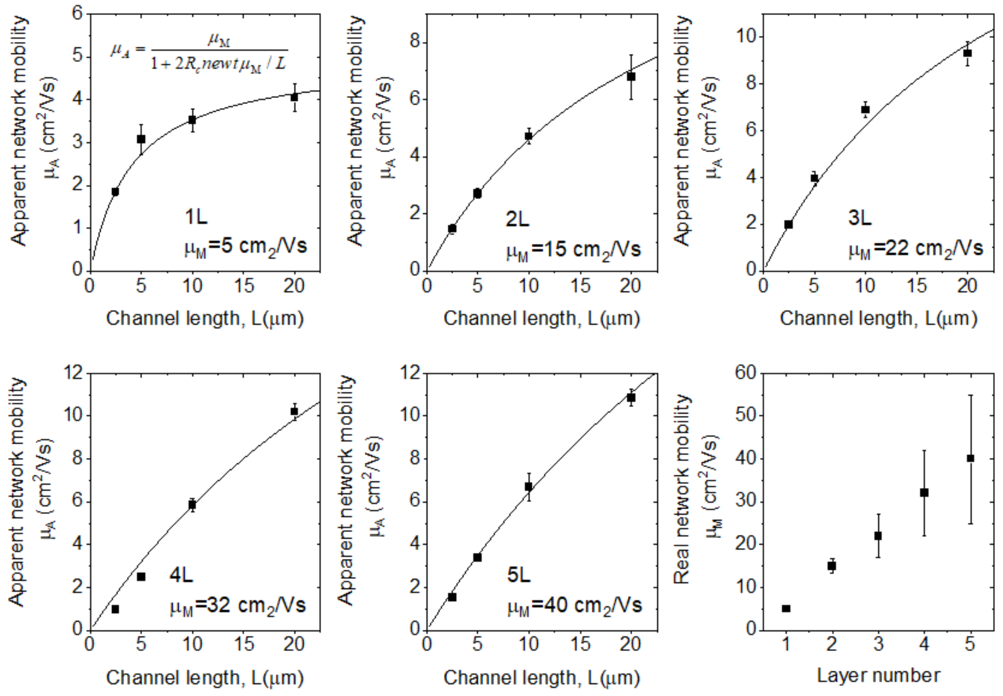

Figure S12: Graphs of measured, apparent network mobilities versus chanel length for five different network thicknesses (1L, 2L, 3L etc). The solid lines are fits to the equation given

above and reprodiced in the top left panel. The bottom right panel shows the resultant mobility as a function of network thickness (1L, 2L etc). The uncertainty increases for thicker films as the data shifts more into the linear regime when L/t is small. In this regime, there is less curvature to the graph leading to greater uncertainty in μ_M.

4: Correcting network mobility for the effects of contact resistance (method 2)

As mentioned above, the mobilities measured in the TFT experiments are affected by the contact resistance and so must be considered as "apparent mobilities". Here we describe a second method by which they might be corrected.

In a TFT, in the linear region when $V_g >> V_d$:

$$I_d = k(V_g - V_T)V_d$$

$$k = \frac{\mu_A C w}{L}$$

Here this equation includes the apparent mobility NOT the real mobility because of the effect of contact resistance.

We can remove this effect as follows. Strictly speaking, V_d is the source-drain voltage the carriers feel which is different from the applied voltage across source-drain, V_A, because of the effects of contact resistance: $V_d = V_A - 2R_c I_d$ (this is just subtracting the voltage drops across both contacts from the applied voltage).

Then the current is

$$I_d = k(V_g - V_T)(V_A - 2R_c I_d)$$

This can be rearranged:

$$\left(\frac{V_A}{I_d} - 2R_c\right)^{-1} = k(V_g - V_T)$$

We can call the term on the left the contact resistance corrected conductance, G_c. it is essentially the source drain current divided by the source drain voltage corrected for contact resistance:

$$G_c = \left(\frac{V_A}{I_d} - 2R_c\right)^{-1} = \left(\frac{I_d}{V_d}\right)_{\text{Contact resistance corrected}}$$

G_c can then be plotted versus gate voltage and analysed using:

$$G_c = k(V_g - V_T) = \frac{\mu_M C w}{L}(V_g - V_T)$$

We have calculated G_C using the contact resistance values reported in figure S10 and plotted it versus V_g for each of our transfer curves (Figure S13). All show linear behaviour consistent with a well-defined slope.

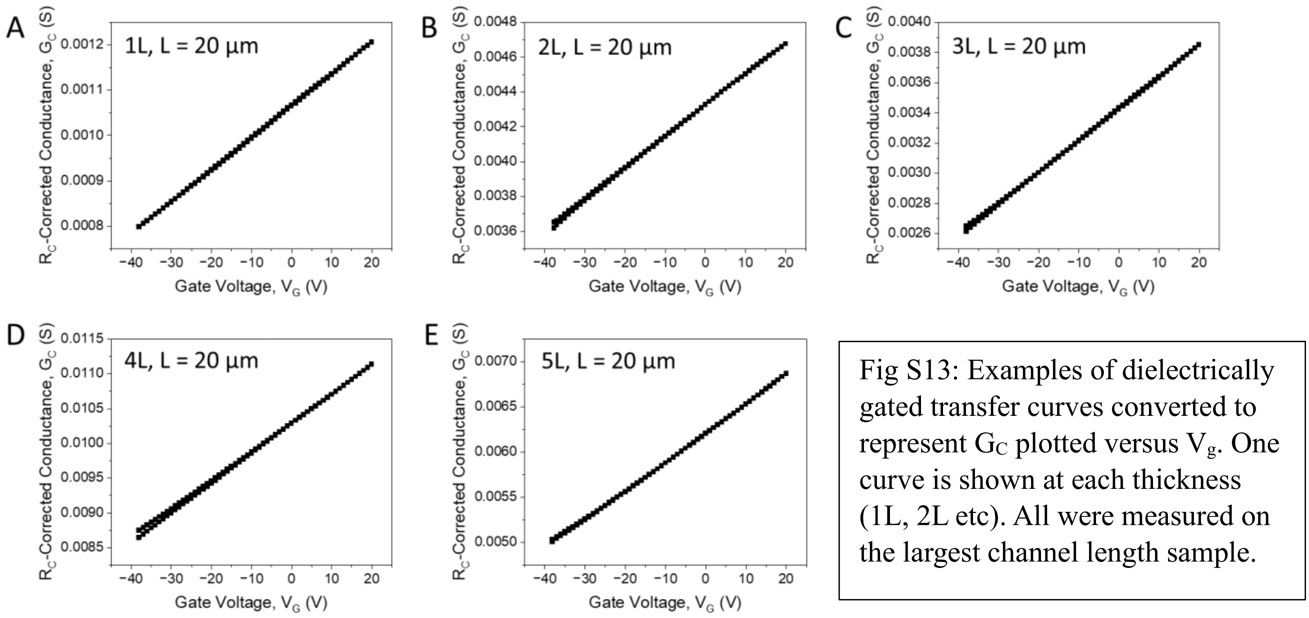

Fig S13: Examples of dielectrically gated transfer curves converted to represent G_C plotted versus V_g. One curve is shown at each thickness (1L, 2L etc). All were measured on the largest channel length sample.

According to the equation derived above ($G_c = \frac{\mu_M C w}{L}(V_g - V_T)$), the contact resistance corrected, real material mobility can be obtained from the slope of the G_c vs. V_g graphs: $\frac{dG_c}{dV_g} = \frac{\mu_M C w}{L}$. This equation predicts that the slope of the G_c vs. V_g graphs should be directly proportional to the inversely channel length, 1/L.

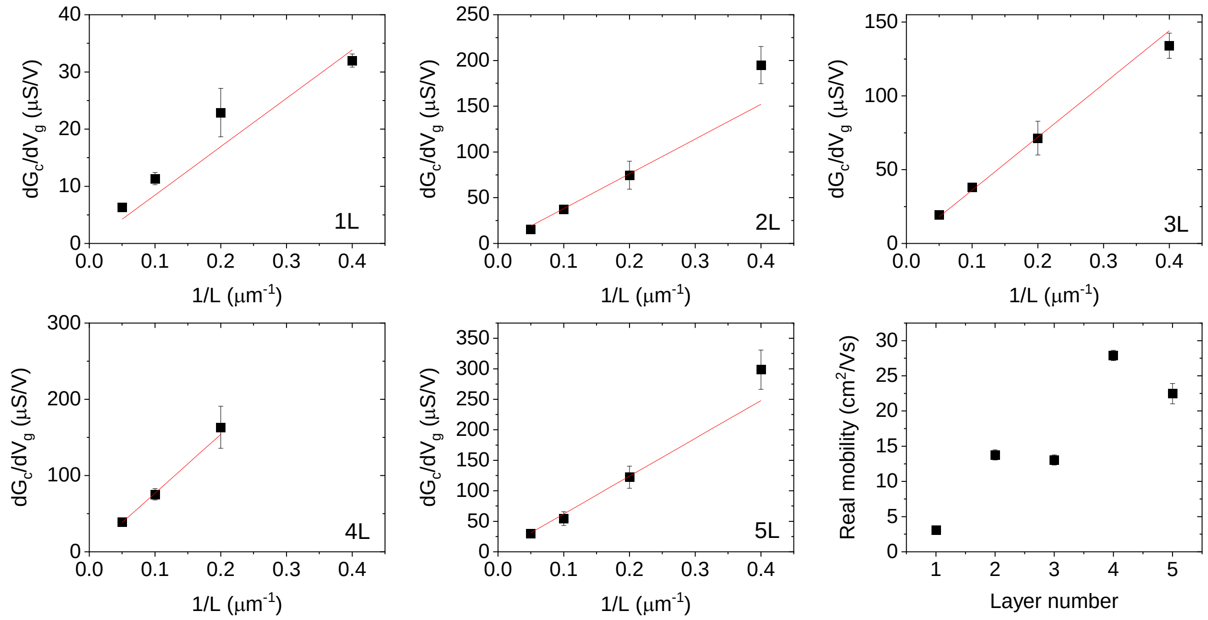

Figure S14: Graphs of dG_C/dV_g vs. 1/L for each network thickness (1L, 2L, 3L, 4L, 5L). Bottom right: the resultant mobility plotted versus layer number.

We have plotted dG_C/dV_g vs. 1/L in figure S14, finding reasonable linearity. From the slope of these graphs, we can obtain the Rc-corrected mobility ($slope = \mu_M C w$). The resultant values are shown in figure S14 and given in table S4.

We take the average of the material mobility values found using both of the above methods. A table of calculated material mobility values is presented below.

Table S4: Measured mobility and material mobility calculated using the above methods.

Layer number	Apparent Mobility (cm^2/Vs) (Rc not removed, 20 μm devices)	Material Mobility from method 1 (cm^2/Vs)	Material Mobility from method 2 (cm^2/Vs	Material Mobility (cm^2/Vs) (Avg methods 1 and 2)	Material Mobility_SD (cm^2/Vs)
1	4.0	5	3	4	1.4
2	6.8	15	14	14.5	1
3	9.3	22	13	17.5	6
4	10.2	32	28	30	3
5	10.9	40	22	31	12

REFERENCE LIST

(1) Miao, F.; Majee, S.; Song, M.; Zhao, J.; Zhang, S.-L.; Zhang, Z.-B. Inkjet printing of electrochemically-exfoliated graphene nano-platelets. *Synthetic Metals* **2016**, *220*, 318-322.
(2) Liu, B.; Zhang, Q.; Zhang, L.; Xu, C.; Pan, Z.; Zhou, Q.; Zhou, W.; Wang, J.; Gu, L.; Liu, H. Electrochemically Exfoliated Chlorine-Doped Graphene for Flexible All-Solid-State Micro-Supercapacitors with High Volumetric Energy Density. *Advanced Materials* **2022**, *34*, 2106309.
(3) Marković, Z. M.; Budimir, M. D.; Kepić, D. P.; Holclajtner-Antunović, I. D.; Marinović-Cincović, M. T.; Dramićanin, M. D.; Spasojević, V. D.; Peruško, D. B.; Špitalský, Z.; Mičušik, M.; et al. Semi-transparent, conductive thin films of electrochemical exfoliated graphene. *RSC Adv.* **2016**, *6*, 39275-39283.
(4) Gabbett, C.; Doolan, L.; Synnatschke, K.; Gambini, L.; Coleman, E.; Kelly, A. G.; Liu, S.; Caffrey, E.; Munuera, J.; Murphy, C.; et al. Quantitative analysis of printed nanostructured networks using high-resolution 3D FIB-SEM nanotomography. *Nature Communications* **2024**, *15*, 278.
(5) Parvez, K.; Worsley, R.; Alieva, A.; Felten, A.; Casiraghi, C. Water-based and inkjet printable inks made by electrochemically exfoliated graphene. *Carbon* **2019**, *149*, 213-221.
(6) Majee, S.; Song, M.; Zhang, S.-L.; Zhang, Z.-B. Scalable inkjet printing of shear-exfoliated graphene transparent conductive films. *Carbon* **2016**, *102*, 51-57.
(7) Calabrese, G.; Pimpolari, L.; Conti, S.; Mavier, F.; Majee, S.; Worsley, R.; Wang, Z.; Pieri, F.; Basso, G.; Pennelli, G.; et al. Inkjet-printed graphene Hall mobility measurements and low-frequency noise characterization. *Nanoscale* **2020**, *12*, 6708-6716.
(8) Pan, K.; Fan, Y.; Leng, T.; Li, J.; Xin, Z.; Zhang, J.; Hao, L.; Gallop, J.; Novoselov, K. S.; Hu, Z. Sustainable production of highly conductive multilayer graphene ink for wireless connectivity and IoT applications. *Nat Commun* **2018**, *9*, 5197.
(9) Clifford, K.; Ogilvie, S. P.; Amorim Graf, A.; Wood, H. J.; Sehnal, A. C.; Salvage, J. P.; Lynch, P. J.; Large, M. J.; Dalton, A. B. Emergent high conductivity in size-selected graphene networks. *Carbon* **2024**, *218*, 118642.
(10) Neilson, J.; Avery, M. P.; Derby, B. Tiled Monolayer Films of 2D Molybdenum Disulfide Nanoflakes Assembled at Liquid/Liquid Interfaces. *ACS Appl. Mater. Interfaces* **2020**, *12*, 25125-25134.
(11) Joung, S.-Y.; Yim, H.; Lee, D.; Shim, J.; Yoo, S. Y.; Kim, Y. H.; Kim, J. S.; Kim, H.; Hyeong, S.-K.; Kim, J.; et al. All-Solution-Processed High-Performance MoS2 Thin-Film Transistors with a Quasi-2D Perovskite Oxide Dielectric. *ACS Nano* **2024**, *18*, 1958-1968.
(12) Ippolito, S.; Kelly, A. G.; Furlan de Oliveira, R.; Stoeckel, M.-A.; Iglesias, D.; Roy, A.; Downing, C.; Bian, Z.; Lombardi, L.; Samad, Y. A.; et al. Covalently interconnected transition metal dichalcogenide networks via defect engineering for high-performance electronic devices. *Nature Nanotechnology* **2021**, *16*, 592-598.
(13) Lin, Z.; Liu, Y.; Halim, U.; Ding, M.; Liu, Y.; Wang, Y.; Jia, C.; Chen, P.; Duan, X.; Wang, C.; et al. Solution-processable 2D semiconductors for high-performance large-area electronics. *Nature* **2018**, *562*, 254-258.
(14) Tian Carey, A. A., Luca Anzi, Helen Bristow, Fei Hui, Sivasambu Bohm, Gwenhivir Wyatt-Moon, Andrew Flewitt, Andrew Wadsworth, Nicola Gasparini, Jong M. Kim, Mario Lanza, Iain McCulloch, Roman Sordan,* and Felice Torrisi*. Inkjet Printed Circuits with 2D Semiconductor Inks for High-Performance Electronics. *Advanced Electronic Materials* **2021**, *7*.
(15) Carey, T.; Cassidy, O.; Synnatschke, K.; Caffrey, E.; Garcia, J.; Liu, S.; Kaur, H.; Kelly, A. G.; Munuera, J.; Gabbett, C.; et al. High-Mobility Flexible Transistors with Low-Temperature Solution-Processed Tungsten Dichalcogenides. *ACS Nano* **2023**, *17*, 2912-2922.
(16) Lee, Y.-I.; Kim, S.; Jung, S.-B.; Myung, N. V.; Choa, Y.-H. Enhanced Electrical and Mechanical Properties of Silver Nanoplatelet-Based Conductive Features Direct Printed on a Flexible Substrate. *ACS Applied Materials & Interfaces* **2013**, *5*, 5908-5913.
(17) Stewart, I. E.; Kim, M. J.; Wiley, B. J. Effect of Morphology on the Electrical Resistivity of Silver Nanostructure Films. *ACS Applied Materials & Interfaces* **2017**, *9*, 1870-1876.
(18) Tai, Y.-L.; Yang, Z.-G. Preparation of stable aqueous conductive ink with silver nanoflakes and its application on paper-based flexible electronics. *Surface and Interface Analysis* **2012**, *44*, 529-534.

(19) Kelly, A. G.; O'Reilly, J.; Gabbett, C.; Szydłowska, B.; O'Suilleabhain, D.; Khan, U.; Maughan, J.; Carey, T.; Sheil, S.; Stamenov, P.; Coleman, J. N. Highly Conductive Networks of Silver Nanosheets. *Small* **2022**, *18.*
(20) Jeong, S.; Song, H. C.; Lee, W. W.; Choi, Y.; Ryu, B.-H. Preparation of aqueous Ag Ink with long-term dispersion stability and its inkjet printing for fabricating conductive tracks on a polyimide film. *Journal of Applied Physics* **2010**, *108*.
(21) Li, X., Sun, R., Guo, H., Su, B., Li, D., Yan, X., Liu, Z., Tian, J. Controllable Doping of Transition-Metal Dichalcogenides by Organic Solvents. *Advanced Electronic Materials* **2020**, *6*, 1901230.
(22) Schindelin, J.; Arganda-Carreras, I.; Frise, E.; Kaynig, V.; Longair, M.; Pietzsch, T.; Preibisch, S.; Rueden, C.; Saalfeld, S.; Schmid, B.; et al. Fiji: an open-source platform for biological-image analysis. *Nature Methods* **2012**, *9*, 676-682.
